# Personal growth initiative across the life span: a systematic review protocol of quantitative studies using the Personal Growth Initiative Scale-II

**DOI:** 10.1186/s13643-024-02546-9

**Published:** 2024-05-09

**Authors:** Katleen Verdoodt, Marianne Simons, Natascha de Hoog, Jennifer Reijnders, Nele Jacobs

**Affiliations:** 1 Faculty of Psychology, Open Universiteit, P.O. Box 2960, 6401 DL Heerlen, The Netherlands; 2 Department of Psychiatry and Psychology, School for Mental Health and Neuroscience, Maastricht University Medical Centre, Maastricht, The Netherlands

**Keywords:** Personal growth initiative, PGIS-II, Protocol, Systematic review

## Abstract

**Background:**

Personal Growth Initiative (PGI) a multi-dimensional construct, conceptualised as a skill set that helps individuals to intentionally grow is considered an important construct throughout the life span. Coping with the challenges, transitions, experiences and stressors of life requires an active growth orientation. In previous empirical research, the construct has been measured by either the PGIS-I or PGIS-II, of which only the latter takes account of the theoretically established multi-dimensionality of the construct. This paper describes the protocol for conducting a systematic review of published peer-reviewed empirical research articles on the multi-dimensional construct of PGI. The aim of this review is threefold: (1) to better understand the multi-dimensional construct PGI in different contexts and populations, (2) to improve our understanding of the reliability and validity of the PGIS-II in various research populations and (3) to obtain an overview of its associations with relevant psychosocial factors.

**Methods:**

For the development of this protocol, the Preferred Reporting Items for Systematic Reviews and Meta-Analyses Protocols (PRISMA-P) reporting guidelines were used. Four databases and one registry will be searched using a predetermined search strategy for relevant studies. Studies will be screened, by two reviewers independently, against the established inclusion criteria. During the data extraction process, the quality of the included studies will be assessed using the Quality Assessment for Survey Studies in Psychology (Q-SSP). The collected data will then be analysed and reported in both narrative and tabular form according to the PRISMA 2020 statement guidelines and flow diagram.

**Discussion:**

The findings of this study will increase our understanding of the dynamics of PGI throughout the lifespan, its associations with other psychosocial factors and the psychometric properties of the PGIS-II. It will also clarify where additional research is needed. The objectives of the proposed review can provide a basis for the development of practical applications and interventions.

**Systematic review registration:**

PROSPERO CRD42022377342.

**Supplementary Information:**

The online version contains supplementary material available at 10.1186/s13643-024-02546-9.

## Background

From a lifespan perspective, personal growth is considered to be essential for a well-functioning individual [1] and found to be a key element for maintaining and enhancing well-being and positive psychological functioning throughout the different life stages [2, 3]. Growth can occur without the awareness of the individual, which is often the case in biological growth processes, or entail a conscious process as a result of an important life event [4]. Intentional growth occurs when the individual is not only aware of the changes but also takes active action to achieve growth [5]. The concept of Personal Growth Initiative (PGI), related to the latter, is described as conscious and active involvement in personal growth processes [1] and is considered to be a multi-dimensional construct composed of cognitive and behavioural components [1, 6]. The cognitive component entails knowledge, beliefs, attitudes and values that foster personal growth. The implementation of the cognitive component in conjunction with decisions to actively make changes constitute the behavioural component [7]. PGI can be seen as a range of skills that assist individuals to engage in self-change across different life domains [6].

To further empirically explore the concept of PGI, Robitschek [1] developed the Personal Growth Initiative Scale (PGIS). Although the observed psychometric properties were sufficient, the original PGIS has two major shortcomings. First, not all items included are intrinsic to intentional personal growth [8]. Second, PGI is measured as a one-dimensional construct [1] and, consequently, no distinction is made between the previously established cognitive and behavioural components of the concept [8]. Subsequently, Robitschek et al. [8] developed the multi-dimensional Personal Growth Initiative Scale-II (PGIS-II), which to the best of our knowledge, currently remains the only scale that measures PGI as a multi-dimensional construct. The scale has four correlated factors, namely: readiness for change, planfulness, using resources and intentional behaviour. The cognitive component comprises readiness for change and planfulness. The behavioural component is constituted by using resources and intentional behaviour. With its multifactor structure, this scale provides a more profound measurement of PGI. The influence of each factor on growth efforts can be determined, as can the relationship with psychosocial factors. In addition to individual differences, population-level variations may also occur [8]. Cultural background can be influential as there are cultural differences in growth motivation and psychological desire to grow [6].

The PGI concept was developed from counselling practice and is consistent with the humanistic view of personal growth as a way of approaching life [1]. Active interest in self-change, therefore, differs from the recognition that change is inherent to human development [8]. PGI shows similarities with the tendency toward self-actualization by focusing on growth orientation but emphasises the individual’s active and intentional involvement in changing and developing as a person. PGI skills can be present throughout the entire course of life [6]. By encouraging active growth in various life domains [8] and providing the skills needed for a productive and fulfilling life [1], PGI contributes to the optimal functioning of the individual and belongs to the essence of eudemonic well-being [9] where the actualisation of human potential is key [10]. As such, research has shown that higher levels of PGI are associated with greater well-being [9, 11] and PGI has proven to be an antecedent of well-being [1]. Meanwhile, positive relationships have also been found with other psychosocial factors such as assertiveness [8], life satisfaction [12, 13] and positive affect [12].

In summary, PGI is an important construct throughout the life course. Coping with the challenges, transitions, experiences and stressors of life requires an active growth orientation [8]. A systematic review of research on PGI, as a multi-dimensional construct, may contribute to a better understanding of the dynamics of PGI across the lifespan.

A previous review conducted in 2016 [14] included many studies using the PGI-I scale, studying PGI as a one-dimensional rather than a multi-dimensional construct. The variation in research populations of the studies included in this review was limited, as most of the studies of personal growth during the period covered by this review involved student populations [14, 15]. The current paper describes a protocol for a systematic review that focuses on studies that measured PGI as a multi-dimensional construct and thus covers the period from 2012, when the PGIS-II was developed, to the present day. According to Weigold et al. [16], several more recent studies have addressed population diversification, finding associations between PGI, well-being and related factors in varying life stages and across populations. As a result, this review may provide a better understanding of the dynamics of PGI over the course of life.

### Study objectives

The aim of this review is to better understand the multi-dimensional construct PGI across the life span and in different populations, its measurement and its importance for mental well-being. To achieve this, three objectives were formulated. The first objective, exploring the different research populations in which PGI has been studied, will help to better understand the dynamics of PGI throughout the lifespan. The second objective concerns the psychometric characteristics of the PGIS-II and will improve our understanding of the reliability and validity of this scale in various research populations. The third objective, examining the associations found with psychological factors and well-being, will also enhance the understanding of PGI as a construct across the life course. This will complement findings from the earlier mentioned review [14], which included several early studies using the PGIS-II and found some evidence for the stability of the four-factor structure of the PGIS-II, albeit inconsistently.

Corresponding with the formulated objectives, the proposed systematic review will address the following questions:

- In which different contexts and populations has PGI been studied?
- What are the psychometric properties of the PGIS-II in different research populations?
- What psychosocial factors have been found to be related to PGI across the lifespan?

Although several psychosocial factors have been identified in the earlier review [14] and meta-analysis [16] among student populations, such as self-efficacy [17], psychological adjustment [17], depressive symptoms and rumination [18], the psychosocial factors will not be specified in advance in this protocol. More recent studies including other research populations may focus on a variety of psychosocial factors, which may have not yet been addressed in previous research. In the next section, the method of the proposed systematic review is discussed in more detail.

## Methods

### Protocol design

This systematic review protocol uses the Preferred Reporting Items for Systematic Reviews and Meta-Analyses Protocols (PRISMA-P) reporting guidelines [19] as the conceptual framework (see Additional file 1).

### Eligibility criteria

The systematic review will include quantitative peer-reviewed studies using the PGIS-II scale and published in the English language from 2012. All ages, across populations and settings, will be included to provide a lifespan perspective on PGI as measured with the PGIS-II. Studies will be excluded if (a) the full text is not available (b) no sufficient details are provided about the intended population (e.g. age, gender, cultural background,…) or the psychometric qualities of the scale or correlations with psychosocial factors are missing (authors will be contacted in case of insufficient details). The number of excluded studies will be recorded at each stage with the reason for exclusion.

To answer the first research question, reported data contains the general characteristics of the included studies and details of the population being studied such as age, gender, type of respondents (e.g. general population, students,..), and cultural background. To answer the second research question, the data items of interest comprise the identified factor structure with fit indices and the internal consistency and test–retest reliability of both the total scale and the subscales. Data in validation studies concerning correlations with other constructs and measurement scales will provide evidence for the concurrent, convergent and discriminant validity of the PGIS-II. Also, data regarding measurement invariance of the scale across samples will be captured. Finally, the data items of interest for the third research question consist of all reported psychosocial factors (e.g. well-being, life satisfaction, psychological distress,…) and their correlations with the overall PGI score and the PGI dimensions.

### Search strategy

An extensive search will be performed in the following databases and registers: EBSCO host, PubMed and Web of Science. Two publisher databases will also be searched, namely ScienceDirect and Wiley online library. Additional snowball and citation searches will be performed on the reference lists of the eligible studies. New identified articles that meet the eligibility criteria will also be included. The published literature will be systematically searched using predefined search terms combined with the Boolean operator “OR”. The search terms for the title, abstract and body text are ‘Personal Growth Initiative’ and ‘PGIS-II’. A preliminary search of the literature in the Pubmed database revealed that the search term 'PGI' produced an excess of irrelevant results. Apparently, the abbreviation PGI has different meanings and is also used in medical research literature. Consequently, it will not be used in the final searches. The elaborated search strategy for the PubMed database in (Table 1) will be adapted for use in the other databases and registries.

**Table 1:** PubMed search strategy

("Personal Growth Initiative"[Text Word]) OR ("Personal Growth Initiative"[Title/Abstract]) OR ("PGIS-II"[Title/Abstract]) OR ("PGIS-II"[Text Word])	

### Study screening and selection

The results of the systematic searches will be transferred to Zotero [20], an open-source bibliographic software manager, where duplicates will be discarded. Studies will then be screened for eligibility based on title and abstract according to the inclusion and exclusion criteria. All identified studies will also be screened independently by a second reviewer. Discrepancies will be cleared through discussion or third-party consultation. Next, the full text of the potentially eligible studies will be examined. Finally, the reference list of the included studies will be checked to find additional studies. To include these additional studies the full text will be screened by the two reviewers. The screening process will be presented according to the PRISMA flow diagram [21].

### Data collection process

Two reviewers will perform data extraction using a Microsoft Excel data extraction sheet developed for this review. A codebook provides guidance for completing the extraction sheet. Before starting the final data extraction, a trial will be conducted to check for consistency among the reviewers. Discrepancies between the reviewers will be resolved through discussion or third-party consultation. To finalise the data extraction, the reviewers will compare the completed datasheets and produce a definitive datasheet by mutual agreement. The extracted data will include title, author, year, country, characteristics of the research populations, psychometric qualities and factor structure of the PGIS-II and significant correlations with examined psychosocial factors. The data extraction sheet can be refined by the reviewers, during the data extraction, to ensure the suitability and usability of all the fields.

### Risk of bias in individual studies

To assess the quality of the included studies, the Quality Assessment for Survey Studies in Psychology (Q-SSP) [22] will be used. The Q-SSP is a 20-item checklist that offers the possibility of assessing the methodological quality of the key elements of a survey study. A separate tab in the Excel file for data extraction is provided for the quality assessment. To ensure sufficient quality of the included studies, 70% is adopted as the threshold for the current review, which is in line with the guidelines of the Q-SSP checklist (Protogerou and Hagger, 2020) If the quality is considered to be inferior, the reason will be stated in the comments on the datasheet. Methodological limitations that have been identified will be considered in the discussion of the results and conclusions. The quality of the included studies will be rated independently by two reviewers. Inter-rater reliability will be checked for consistency and discrepancies will be cleared through discussion or third-party consultation.

### Data synthesis, presentation and dissemination of findings

The objective of this systematic review is to provide a comprehensive overview of the studies on PGI using the PGIS-II as a measurement, the populations in which it is measured, the psychometric qualities of the scale and the relationships found with psychosocial factors. Therefore, a synthesis of the results will be reported in both narrative and tabular form. To write up the final systematic review the PRISMA 2020 statement guidelines and flow diagram [19] to report on the search findings will be followed. The review will be submitted for publication in an international peer-reviewed journal.

## Discussion

Throughout the life course, personal growth can be considered an important component of optimal functioning and well-being. The earlier review, discussed in the introduction [14], has provided a useful starting point for a better understanding of the concept of PGI and the contribution of PGI skills to intentional growth moments. A previously conducted meta-analysis [16] has shed light on several psychosocial factors related to PGI. The current review explicitly focuses on PGI as a multi-dimensional construct and hopes to include studies that vary in research populations, in order to clarify the extent to which PGI has been studied across different life stages and cultural backgrounds and where additional research is needed. The stability of the multifactorial measurement scale when used across populations also merits additional attention. In addition, the objectives of the proposed review may also provide a basis for the development of practical applications and interventions. Uncovering associations between PGI and other factors, throughout different life stages, can help design targeted interventions to promote PGI.

By using the PRISMA guidelines [19, 21] to shape this protocol, a solid framework for research was created. However, by including only peer-reviewed studies, publication bias is a risk. Consequently, such bias can be seen as a limitation of this review.

### Supplementary Information

**Additional file 1.** PRISMA-P 2015 Checklist.

## Data Availability

Not applicable.

## Abbreviations

PGI: Personal Growth Initiative
PGIS-II: Personal Growth Initiative Scale-II
PRISMA-P: Preferred Reporting Items for Systematic Reviews and Meta-Analyses–Protocols
PRISMA 2020: PRISMA 2020 explanation and elaboration: updated guidance and exemplars for reporting systematic reviews
PROSPERO: International prospective register of systematic reviews
Q-SSP: Quality Assessment for Survey Studies in Psychology
